# Something on Medical Mistakes

**Published:** 1896-12

**Authors:** B. E. Hadra

**Affiliations:** San Antonio, Texas


### TEXAS MEDICAL JOURNAL.

ESTABLISHED JULY, 1885.

PUBLISHED MONTHLY.—SUBSCRIPTION $2.00 A YEAR.

VOL. XII. AUSTIN, DECEMBER, 1896. NO. 6.

### Original Contributions.

For the Texas Medical Journal.

### SOMETHING ON MEDICAL MISTAKES.

BY DR. B. E. HADRA, SAN ANTONIO, TEXAS.

[Annual address of the outgoing President of the West Texas Medical Association, October 29, 1896.]

IT IS the duty of the outgoing president to enlighten the society with an address; but I deem it best not to belabor you with a profound scientific discourse, which, I am sure, would hardly be enjoyed by you, after having already listened to so many grave papers. I consider it more desirable to offer my contribution, rather as an appetizer for the after-coming banquet, at which, I hope, you will be indemnified for your toilsome work of to-day; and the longer my paper, the more will your digestive juices accumulate. Permit me, then, to make a few unpretentious remarks on a theme, ever interesting; namely, on medical mistakes, with especial reference to those in diagnosis.

It is an *old truth* that we can learn more from our mistakes than from our success. It is evident that we knew less and were not so well informed on thsse matters in which we erred; hence, we are made to realize our defects the more markedly and durably, though not so pleasantly.

I do not believe that I need to apologize for speaking unreservedly, "fresh from the liver," as the German would say, because I hope that I do not address myself to anyone who has never made a mistake. But, if by chance, one such exalted personage should be found in our midst, he has my permission to publish broadcast my exposure of medical misdeeds and medical outrages.

In fact, the more various the cases are that a medical man comes in contact with, and the more he has thought and studied, the easier he may err, for our art is one of possibilities and probabilities, and the larger the number of possibilities which present themselves to one's mind, the easier it will be to make a wrong selection. This is no paradox, and it does not follow that mistakes bear a direct relation to a physician's ability.

The well-informed man will think of possibilities of which the ignorant will not even dream; and while the former will now and then make a mistake through pardonable miscalculation, the other will avoid diagnostic mistakes only by not making any diagnosis at all. As a shining example of such a never-erring doctor, I recall one who, in former years, held the first place in the public estimation of a neighboring city, and who accumulated an exceptionally large fortune. He acted on the simplest conceivable, and at the same time surest principle that human ingenuity and scientific foresight could devise;—he diagnosticated on a strictly anatomical basis. Dividing the human body into five or six districts, and then placing the trouble into one of these divisions, he could not fail in pronouncing the case to be a disease of the head, or of the thorax, abdomen, lower extremities, etc. His infallible diagnosis suited him no less than it did his admiring clients, who could themselves comprehend the correctness of his verdicts.

Gentlemen, I would, for practical purposes, divide our mistakes into such as can be hidden from the public, and such that will be discussed by every old woman for years.

But surely the highest pinnacle of practical medical ability is attained when one can turn his mistakes into sources of public admiration and remuneration. There are a few such artists in every community. These wonderful men generally detect a whole tapeworm of diseases in every case where the stupid, every-day practitioner does not find but one. They, for instance, discover typhoid fever in a case to-day; by to-morrow it has been converted into a child-bed fever; the third day an appendicitis supervenes, which in its turn will be followed by a serious meningitis. Blood poisoning and heart failures are a matter of course, but these acute diagnosticians recognize each new fad at once. They immediately erect bulwarks of defence with as much noise as the Chinese use in their warfare, lay out counter-mines, and then come out victors over all these terrible forces combined, or get overpowered by the alliance of too great a number of adversaries. To these rightfully belong the laurels and the golden crowns, because they are smarter than all the others.

The question whether you should acknowledge your mistakes to the public or not, is, no doubt, a very ticklish and knotty one. Even the greatest thinkers in our profession differ widely on this. One faction pronounces you a fool if you do, prefixing this friendly classification with an adjective, and, as a rule, only in the lowest society, on the stage and in the pulpit. The other side holds diametrically opposite views. It demands of you straight out honesty; it commands you to explain your mistakes even when nobody requests it of you. These watch dogs of morals and ethics, generally, watch everybody but themselves. Now, it is claimed that honesty is the best policy in the long run. But the trouble is, you can never tell in advance how long that run will last. The poor fellow who follows this rule too closely may find himself in the same fix that that celebrated horse did, which was in training to live on nothing, and which, unfortunately, died just then, when it had succeeded in mastering the problem. But let us not hastily shed tears over the fate of such unfortunate colleagues. I have never found one yet;—perhaps he does not exist.

It certainly requires a keen knowledge of human nature to decide to whom or how much of the truth you may confide to the nterested parties. As a rule, the intelligent client will give you credit for your manliness and honesty, and his confidence in you will rather increase, because he can see that you reflect on your cases, and that you are open to improvement. But it would be the height of folly to tell Tom, Dick and Harry that you were mistaken in your diagnosis, perhaps up to the post-mortem. You never would be forgiven, and their hearts would never cease to bleed. You will have done no good to anybody. It is, therefore, one of nature's choicest gifts to the doctor, and if she should have neglected him in this respect, it should be his first aim to acquire a goodly portion of it,—I mean that which the uneducated people generally call cheek or brass, but which is, in reality, the characteristic symptom of the philosopher—that equanimity, that impenetrability of countenance that knows neither consternation nor surprise. The austere, profoundly thoughtful scientist says as little as possible,—every word from his lips ought to be looked upon as a precious gift. Absorbed in the depth of unravelling the case before him, like an Archimedes, he must of necessity be inaccessable to the inquisitiveness of common beings. However, if he cannot avoid making some explanation, he will resort to the next best expedient—he will express his opinion in highly sounding scientific terms, pre-eminently using Greek and Latin. The less able the old women are to understand him the more they will try to appear to do so.

An amusing example of such a philosopher is a very successful practitioner in one of our more sparsely settled counties, where he fills the combined cathedra of a Billroth, a Pepper, a Marion Sims and a number of other specialists. Though I only know him from heresay, I can vividly picture the man before me. Tall, full of graceful dignity, enwrapped in profound thought, adorned with flowing whiskers and an extra tall silk tile; he would, when asked about the condition of his patients, slowly stroke his long whiskers, give his feet a scrutinizing stare, and then remark, with an expression pregnant with the profoundest wisdom, "Too much hebetude!" In fact, there is *hebetude* in every one of his cases, and the amount of this mysterious virus is, in the estimation of his admiring clientelle, the barometer of the patient's condition. "How is his hebetude, doctor?" is the standing question. They die from too much hebetude in that county, but are often saved by this great doctor because he knows how to battle against this terrible hebetude.

But it would be a very one-sided treatise, and a most unworthy effort on the part of an ex-president of your exalted society, if I should only view this great problem from one standpoint. Let me, therefore, suggest another division of medical mistakes, namely: those that are pardonable, and those that are not. I am willing to admit that cases arise where a correct decision under this classification would be hard to make. To prove the correctness of my assertion, I submit the following experience of my own. You may pass judgment yourselves:

Many years ago I was asked by a colored porter, who worked in the same building in which I had my office, to go to see his wife, who was suffering from "some misery in the belly." When I approached the lady's sick-bed, I found her covered with feather beds, it being winter, and I could, with great difficulty, only gain the information between her noisesome grunting, that she had terrible cramps in her stomach. As the atmosphere of her room was not exactly inviting, without much ceremony I prescribed some opiate and left, fully convinced that I had done my duty. The next day I met my colored friend again, but he not only totally ignored me; no, he gave me such an unmistakable look of contempt, that I could not help but feel that indescribable mean guiltiness which will steal over you when about to be confronted with some misdeed already vaguely apprehended. But I was determined to swallow the bitter dose at one gulp. I compelled Sambo to stop, and collecting all my courage I asked him about the condition of his wife. At first his disdain for me seemed to forbid him answering at all, but finally his rage got the better of him, and he burst out like a broken koprostasis: "How my wife am? Ah, yes, you is the doctor, is you? Very fine doctor, you is—belly-ache!—fine belly-ache! She had a baby ten minutes after you left—a fine doctor, you is!" I did not prolong my conversation with the irate father of the new-born. Now, gentlemen, I leave it to you—guilty or not?

But to return to our question—our errors are unpardonable when they are due to unnecessary haste and carelessness. These errors happen mostly in office practice, especially when the office is over-crowded, and when the physician must feel very nervous, as I can testify,—from heresay. Ordinary mortals do not often get into such an uncomfortable situation. Contemptible, or at least puerile, is an intentional rapidity to bluff the patient with a diagnosis or prescription before he has even had time to state his case. It is done with a view to impress the public with one's wonderful gift of penetration, laying even a Roentgen ray apparatus in the shade. In these days, when we are eager to improve the means for examining the hidden parts of the human body, such artifices are so much the more base quackery. It is not necessary to dwell longer upon this kind of kodak diagnosis.

The most common unpardonable, as well as pardonable, mistakes are made, as a matter of course, through ignorance. It will, at times, be impossible to avoid them, simply because certain matters are still in perfect darkness to science. In fact, we should not rob the innumerable generations to follow, of all opportunities for new discoveries. I confess that when I look over the vast number of additions our knowledge has acquired, and when I reflect how little, in spite of all these I now know, and how much less I must have known thirty years ago, I say, I do not see how I could have dared to offer my services as a healer; and still less do I comprehend how I could have given as much satisfaction as I did, to at least a part of my clients. And yet, centuries ago, when perfect darkness, coupled with superstition and quackery, reigned in our art, there were celebrated physicians, to whom mankind flocked from every corner of the world. How is it that the physician could maintain his reputation then, equallly as well as fifty and one hundred years later, to-day; or, in short, as he has done in every age? Is it the same cause that permits the Indian medicine man to flourish, and nothing else? Yes, and no. On the one hand, to some extent, medical art is based on suggestion, credulity, faith and deception; on the other hand, thorough good common sense and good judgment have been the foundation of success, from time immemorial up to the present day, and have, in a measure, determined the superiority of one doctor over the other, as it is the case between men in every other occupation. But we can state with pride, that this century had added so much real knowledge, of such indisputable value in diagnosis and treatment, that well the most conscientious man may feel justified in offering his services as a physician and surgeon without even thinking of depending on dishonorable methods, providing he has grasped the practical results of these new revelations. It would be of great value both to the public and to our profession, if the former could be made to better understand our true position; if it could be impressed with the fact that not everything pertaining to human ailments is known, and that even the best of us must, therefore, occasionally make mistakes. It would be a blessing if the people would look with less distrust upon us, and allow us to be more candid and honest towards them, instead of making us constantly watchful and nervous lest we may drop a word that could be misconstrued. How is it, for instance, that the lawyer is permitted, nay, even expected to consult his authorities before tackling an important case, while a doctor who would admit the necessity of doing likewise would at once be proscribed as an ignoramous, though he has a much vaster field to cover, and is much more dependent upon a good memory.

Still more should such considerations teach physicians to be charitable toward each other; they are certainly *all* sinners. But it is quite a different thing with the quack, the pretender; heshould find no mercy. Our concession that we may and do make mistakes, is certainly no excuse for him who has not even tried to acquire the means to avoid them. We honestly strive to gain much light as science offers, whilst he lives solely on the credulity of the populace, throwing sand in their eyes by imitating our external ways and methods. It is unnecessary to assert that we will err less often than he.

But there is another source of error, more or less pardonable. It is the preocupation of the medical mind, which will sometimes hinder even the ablest of us from forming an unbiased opinion. I suppose we have all experienced such occasions, when an idea, quickly founded on, may be, a very trifling symptom, will prevent us from thinking in any other direction. We will, to our own future wonderment, stick to such an opinion until some disaster, or the kind helping word of a colleague will put us on the right track.

Somewhat akin to these mistakes are those of specialists who view everything from their own standpoint.

But prejudice is often a source of less pardonable mistakes. I mean that outcome of either spite against others, or overestimation of one's ability; it is the *ab initio* contempt for other doctors' opinions and the desire to prove himself the smarter man. Thus, for instance, a certain physician of our place would never allow a diagnosis of mine to pass his exalted throne unchallenged. If a plain fracture should have been made out by me, and if he should be called in to confirm my opinion, he would certainly pronounce it a dislocation, a commotion of the brain, piles, or what not, according to the accidental fierceness of his sentiments. I think this habit is now perfectly automatic with him. Of course such methods will not work very well with intelligent clients, while I concede that they take with the masses. There is, unfortunately, in no other profession such a tendency for one man to consider himself abler than the other man. Some doctors will, *ab initio*, abhor other men's views, throw out their medicine bottles, refuse to listen to their opinion and treatment, and so on. Perhaps it is a necessary stock in trade in our business, to have this self-confidence and self-admiration, in order to do the work with as much dramatic decision, as much self-reliance, and as much autocratic, god-like feeling as the serious task of presiding over the destiny of human welfare and life demands.

Let us now view a more cheerful and congenial picture. It is the most pardonable of all diagnostic mistakes: the intentional error, better expressed as the conscious lie. The pulpit men call it a sin, but these sins will be committed as long as mankind exists: even angels will commit them, when circumstances call for it. They are the outcome, either of compassion and humanity, or are called for by the necessity to direct the patients mind into other channels. In the first place, you will mostly have to deceive the hopelessly sick; but I would advise you to take some near relative into the secret, lest some charitable colleague should have the opportunity to proclaim that he never had seen such ignorance in a doctor. As a general rule though, it is much wiser to lean rather toward a bad prognosis; to alwsys look very grave, and never to forget to shake your head and shoulders a little, but in such a manner that it would appear that you wished to do so unobserved. The omniscient and all-wise old woman will understand you. You will then always be in the most favorable position, for if the patient recovers, you will get that much more credit for the wonderful cure; if, on the other hand, he should prefer to leave this world of woe, well, then you will have known it from the beginning. Another highly commendable method in questionable cases, requiring, however, a great deal of tactical ability—is to state the case in a favorable light to one influential personage, and in a very critical and unfavorable light to another equally important one. You can then, whatever the outcome may be, refer to one of these good witnesses. But do not forget to keep these two people from exchanging their experiences.

Your intentional erroneous opinions, given as a kind of faith-cure, will, as you know, come in mostly in hysterical patients. As an illustration, let me narrate to you an anecdote told on a celebrated Vienna professor, who had to treat a hysterical lady, for whom he had prescribed an indifferent medicine, and whom he had entreated, under no circumstance to take it except between two drinks of water, so that it would be in the middle. She did splendidly under this regime; but one day the professor was summoned in great haste, and found the patient laboring under the greatest excitement. She had forgotten to take the water before the medicine. The professor appeared shocked, but was happy to have been called in time to prevent dangerous consequences. He injected a dose of water into the rectum, and explained to the lady that now the medicine would be in the middle again. This explanation perfectiy quieted her fears.

Such subterfuges are obviously pardonable, if not entirely necessary. They do good to the patient, and fortunately, also to the doctor. Think of the trying situation where you are asked for a strict diagnosis while you are yet in the profoundest darkness about the trouble. You must then draw on your off-hand readiness of invention and lying. It it quite amusing to see what wonderful diseases the fertile medical mind may be able to create. I know, for instance, of such diagnoses as, "shock to the lining membrane of the heart, or the invasion of bacilli into the fourth ventricle, or rheumatism of the spermatic artery, or dislocation of the pneumo-gastric nerve, etc. The danger lies in forgetting what you previously pronounced the case, especially if you are invited next morning by the mother to discuss the case with her. In this connection I am proud of the Faculty of our town. I know some of our practitioners who compete, without fear, against Don Pedrito, who, in my opinion, is a real phenomenon in this line.

But to proceed more seriously; the most pardonable of all mistakes are those where the differential diagnosis lies between one or two things in the proportion of 1:1. We all know on what trifles our choice may depend, and I could never suppress a comparison between our diagnosticating maneuvers and the mental processes active in a sharp game of the great American play,—poker! Successful work depends, in both instances, upon the correct combination of all visible and invisible circumstances; on sharp observation and appreciation of the slightest points that may throw the least ray of light upon the state of things; and all this must be done in the shortest possible time, and in the most encompassing way. In fact, it is the same difference between doctor and doctor as between player and player. In both instances one can at times hardly define the cause of the difference of success between one man and the other. It often looks like a kind of intuition, a mysterious gift, that enables one doctor or player to devine the right thing at the right time, more so than the other; still it is mostly a real superiority in quick and correct perception and judgment, based on a number of factors, which may appear so insignificant that even the doctor or the player himself is not conscious of his extraordinary ability to make use of them. Now, I hope you will not suspect me of advising the fraternity to practice that noble pastime, but allow me to remark that a doctor who plays the great national game poorly, after due course of practice, had better quit doctoring also.

I do not know how to classify a defect, either natural or due to a want of clinical training, which we so often find in medical men, who may be able to recite whole volumes and exhaustive treatises on any topic pertaining to their science, and still be perfectly lost at the bedside, where they will not be able to discern the plainest diseases from each other. All of their brain cells will then be set to work at once, and there will be such a pell-mell confusion of reminiscences that a definite decision cannot be arrived at. If such an inability to differentiate important phenomena from indifferent ones is congenital, we can express nothing but sympathy for that doctor; because he must naturally make more mistakes than we would be willing to allow the average practitioner. And yet his success as a business man would not be greater. His destination is routine work in an officer under somebody's supervision; or quiet, steady work in the library.

There are also confusionists from other causes. I mean those mostly self-made men who have read an immense amount, but are wanting in clinical training. They have no method, and no system to look at diseases. They never did learn to weigh objectively, where the greatest possibility lies. They take accidental signs for the very essence of the trouble, and vice versa. He who has not had such a clinical training for a sufficient length of time, must be a man of extraordinary judgment indeed, to make good this deficiency. It is just this which the school is pre-eminently offering, while positive knowledge must be accumulated greatly in after-school times.

An old, old reminiscence occurs to my mind here, a consultation, to which I, to my utter satisfaction, was summoned, with no less than three other physicians, the most prominent in the town, of course, and where I had to pass sentence over the verdict of an able and clever brother practitioner, concerning a case of plain cancer of the stomach, in the very last stage. One after the other of us stepped forward between two rows of closely watching relatives, to the poor fellow's layer,—one after the other felt pulse, looked at the tongue, grasped the nicely developed tumor in the stomach, and then retired in solemn silence, but with clearly expressed approval of the diagnosis made by the family physician. But when the fourth man's turn came, the scenery and action changed. Obviously, he was the star, so considered by himself and the audience. It was a grand moment when he approached the couch. The well known hacking cough preparatory to undertaking great feats, the cautious, sly movements, like those of a horse trainer not fully trusting the disposition of his beast, then the general survey of the object—it was wonderful. Now, he had the poor patient fully exposed, and after an intense scrutiny of a general character, he began to examine the man, starting at the testicles and going upwards to the stomach, lungs, mouth, eyes, etc. But the heart was the chief object to which he devoted himself, and which he watched with the profound sincerity of an Indian scout. The patient's relatives expressed their admiration for the great doctor, by unmistakeable signs, elbowing each other and nodding their heads in approval, while they treated all the rest of us with studied contempt. I confess that I felt very sheepish and apprehensive for fear that my methods of examination were utterly defective. After half an hour or longer of examination, the great chief, with a graceful movement of his hand, and without uttering a sylable, invited us into an adjoining room for a consultation (pow-wow). Here he surprised us by the short but decisive statement that the patient was suffering from a rent of the right auricle of the heart. I was speechless for several moments; but my youthful indignation broke loose at last, with a very unparliamentary remark; after which I wisely withdrew. I had suspected him of trying to work that old racket of finding something entirely different, when nothing can be proven to the public, and when it is too late for him to do any more good; but I was subsequently informed by other physicians that this extraordinary man made it his rule to examine every patient in the same exhaustive manner, and that it was just this trick which made him so popular and so much sought after as an adviser in his community. No one, though, ever had heard of a correct diagnosis of his. The family of the deceased auricular rent victim never ceased to express their regret at not having called in the great doctor long before, he being the only one who ever knew what the patient was suffering from.

Without wishing to tax your indulgence too much, let me say a word on one more kind of pardonable mistakes, such as are the results of the weakness of human flesh—and God knows—physicians suffer from it, at least as much as other human beings. They are the mistakes of exhaustion, want of sleep, of a tired out or worried mind, not infrequently emanating from corporal or mental sufferings, due perhaps to unfortunate business or family affairs. Then there is that which may make even a strong minded man a superstitious creature, that which one may call a bad day, or a contrary hour, where for unaccountable reasons everything goes wrong.

In order to be exhaustive I would have to enumerate a good many sources and kinds of mistakes, but my sympathy for you is so great that I will place them all in the one category: miscellanies. They are as numerous, and as various as the thoughts and accidents of human life. Let me illustrate it by one example, to show how difficult it would be to classify a great many of these cases.

One of my intimate friends settled as a beginner in a small Silesian town, surrounded by Polish-speaking peasants, whose language he intended to learn as fast as possible. But before he had sufficiently progressed, a Polander consulted him without an interpreter being on hand. Placed in this dilema, the doctor concluded to examine the man from head to foot to discover the seat of trouble. In spite of all kinds of protestations on the part of the man, the doctor succeeded in making him undress himself. Not finding anything wrong, an indifferent medicine was prescribed, which the peasant dubiously accepted, and carried to the druggist. He expressed to the latter his doubts about the doctor's mental equilibrium, narrating the doctor's actions when he came to consult him about his sick child. The druggist, glancing at the prescription, and finding that even a child could not be hurt by the medicine, and also not wishing to give the doctor away, told the man that it was one of the new-fangle methods, and that he should do as directed. The child got well, and the doctor had to do his best to prevent the people from the peasant's neighborhood from sending a grown relative who would immediately undress himself for an examination whenever a child was to be prescribed for.

Now, gentlemen, in conclusion, let me assure you that my aim has been to do some good, even though my remarks may seem frivilous and silly. A little self-irony is sometimes the best philosophy. As a matter of course, our shortcomings ought to be known by us better than by others, and our shortcomings better than our merits.

